# The Hungate1000 prokaryotic culture collection encodes a wide variety of bacteriocins

**DOI:** 10.1128/msystems.00195-26

**Published:** 2026-05-27

**Authors:** David Hourigan, Lorraine Draper, Sinead C. Leahy, Graeme T. Attwood, William J. Kelly, Catherine Stanton, Colin Hill, R. Paul Ross

**Affiliations:** 1 APC Microbiome Ireland, Biosciences Institute, Biosciences Research Institute, University College 8795, Cork, Ireland; 2 School of Microbiology, University College Cork, Cork, Ireland; 3 AgResearch Ltd., Grasslands Research Centre, Palmerston North, New Zealand; 4 New Zealand Agricultural Greenhouse Gas Research Centre (NZAGRC) 553141, Palmerston North, New Zealand; 5 Teagasc Food Research Centre, Moorepark 34344 , Fermoy, Co. Cork, Ireland; Fluxus Inc., Sunnyvale, California, USA

**Keywords:** bacteriocin, rumen, bacteria, prokaryote

## Abstract

Bacteriocins are antimicrobial peptides/proteins that are widely distributed among bacteria and are gathering traction as natural alternatives to antibiotics, modulators of the microbiota, and interbacterial signaling peptides. The Hungate1000 is a culture collection of isolated prokaryotic microorganisms and their genomes from ruminant animals that aims to expand the knowledge base of rumen ecology. In this study, 410 rumen-isolated prokaryotes within the collection were mined to expand upon the bacteriocin-producing potential of the rumen. A total of 408 novel bacteriocin gene clusters were identified across 308 genomes. Bacteriocins in novel species within the Hungate1000 were identified, such as *Pseudobutyrivibrio* sp. UC1225, which has two novel natural nisin variants, *Clostridium* sp. DSM 8431 with a novel peptide 81% identity to amylocyclicin and *Lachnobacterium* C7 encoding a novel circular bacteriocin with 55% identity to the circular bacteriocin NKR-5-3B. A novel class II lanthipeptide gene cluster was also identified containing eight distinct core peptides encoded within the genome of a novel *Butyrivibrio* species. Bacteriocin biosynthetic potential was noted within species unknown to produce bacteriocins, such as *Lachnobacterium bovis* DSM 14045, *Lachnospira multipara* D15d, *Eubacterium callanderi* NLAE-zl-G225, *Eisenbergiella tayi* NLAE-zl-G231, and *Muricomes contorta* NLAE-zl-C134. The frequency of putative bacteriocin production within ruminal strains was 30%, doubling the frequency previously suggested in the mammalian gastrointestinal tract. This number increases to ~70% when encompassing groups of peptides with limited knowledge of antibacterial activity, such as ranthipeptides and auto-inducing peptides. We also show that the bacteriocin core peptides mined from the Hungate1000 culture collection are found in the microbiomes of other ruminant animals and the human gut microbiome. These findings highlight the Hungate1000 as a rich biosynthetic reservoir of cultured strains that can be experimentally explored for functional antimicrobial activity. The presence of diverse bacteriocin-producing lineages in rumen-associated microbes provides a foundation for future strategies aimed at targeted microbiome modulation, including approaches to improve rumen function and potentially mitigate enteric methane emissions using bacterial strains or their natural products.

**IMPORTANCE:**

Bacteriocins are gathering traction as a possible alternative to antibiotics in some instances. Therefore, it is crucial to discover novel bacteriocins to expand the bacteriocin knowledge base if these peptides are to be translated to the clinic for use in humans or developed as veterinary interventions to modulate rumen function. Here, we use *in silico* methods to identify the biosynthetic potential of the Hungate1000 culture collection of rumen bacterial strains. We discover 408 novel bacteriocin gene clusters across 308 genomes and identify that the frequency of bacteriocin gene clusters is over 30%, which is double the incidence rate from previous studies of the mammalian gastrointestinal tract. This number increases to approximately 70% when including bacteriocin classes such as ranthipeptides and cyclic-lactone-autoinducer peptides. Together, these findings position the rumen microbiome as a rich and underexplored reservoir of antimicrobial diversity, with potential for the development of targeted microbiome-modulating therapeutics, livestock interventions aimed at improving rumen function, and strategies aligned with One Health goals, including antimicrobial stewardship and methane mitigation.

## INTRODUCTION

Bacteriocins are small ribosomally-synthesized antimicrobial proteins that often have antimicrobial activity against strains similar to the producing organism. They are natural products that have antibacterial properties against closely related species (narrow spectrum) or unrelated species (broad spectrum). They have gathered much interest as natural alternatives to antibiotics due to their reduced collateral damage to the microbiome and efficacy against multi-drug-resistant pathogens (1). Bacteriocins can be categorized as Class I modified or Class II unmodified based on the presence or absence of post-translational modification to the core peptide. Class I bacteriocins encompass an increasingly diverse subgroup of ribosomally-synthesized and post-translationally modified peptides (RiPPs) below 10 kDa (2, 3). These peptides are initially synthesized as longer precursor peptides, generally containing an N-terminal leader responsible for translocation and recognition domains to guide post-translational modification (PTM). This results in an exported mature core peptide often with antimicrobial activity. A table of essential biosynthetic gene cluster (BGC) class-specific biosynthetic proteins is summarized in Table 1. The most extensively studied RiPP subgroup of bacteriocins is the lantibiotics (lanthionine-containing). Nisin is a lanthipeptide initially isolated from fermented milk cultures in 1928 and is currently used as a food preservative. Natural nisin variants have been found in different microbial niches, including the mammalian gut and skin microbiome (4–6).

**TABLE 1:** Rules used to identify putative RiPPs/bacteriocins

Classification	Rule	Count
Ranthipeptides	TIGR03973	224
Cyclic-lactone-autoinducer	TIGR04223	75
RiPP-like	Fall-back rule	71
Class II lantibiotics	LANC_like and DUF4135	30
RRE-containing	ANY_RRE	22
Lassopeptides	(PF13471 and Asn_synthase or micJ25 or mcjC)	21
Class I lantibiotics	(LANC_like and (Lant_dehydr_N or Lant_dehydr_C)) and not all_YcaO	11
Thiopeptide	((YcaO or TIGR03882) and ((thio_amide and (PF06968 or PF04055 or PF07366)) or Lant_dehydr_C or Lant_dehydr_N or PF07366 or PF06968 or PF04055) or thiostrepton)	11
Sactipeptides	(Subtilosin or thuricin or TIGR04404)	4
RaS-RiPP	(PF04055 and TIGR01716)	2
Proteusin	Proteusin	2
Class III-IV lantibiotics	micKC or (LANC_like and Pkinase) and not micKC	1
LAP	((Goadsporin_like or PF00881 or TIGR03605) and (YcaO or TIGR03882))	1
Sonorensin	PF27784	1
Total		476

Class II bacteriocins are generally <10 kDa in size, are heat-stable peptides, and include pediocin-like bacteriocins, unmodified two-component peptides, and linear, non-pediocin-like peptides (7). Prediction of unmodified (Class II) bacteriocins can be supported by detection of the precursor gene and/or by conserved biosynthesis-associated context genes (e.g., transport, processing, and immunity) even in the absence of modification enzymes. However, discovery remains challenging due to limited and incomplete understanding of Class II bacteriocin biosynthetic pathways (8). Pediocin-like peptides have been observed in a broad range of lactic acid bacteria (LAB), which have been isolated from dairy, fermented foods, and the mammalian GIT (9). Unmodified two-component bacteriocins are a diverse group of bacteriocins that can have activity both alone and in a synergistic manner.

The rumen microbiome represents a dense community of bacteria with up to 10^11^ cells/mL that exist symbiotically with the host to provide nutrients in the form of volatile fatty acids such as propionate, butyrate, and acetate (10). Competition for microbial-derived resources has been the premise for searching for antimicrobial peptides (AMPs) and bacteriocins (11–13). Hence, the rumen microbiome has been shown to be a fruitful source of novel antimicrobials, such as bovicin 255, produced by *Streptococcus gallolyticus*, butyrivibriocin AR10 from *Butyrivibrio fibrisolvens* AR10, and butyrivibriocin OR79A from *Butyrivibrio fibrisolvens* OR79 (14–16). *In silico* searching for bacteriocins in 229 bacteria identified bacteriocin gene clusters in the rumen at a frequency below 15%, which mirrors the frequency previously found in the human GIT (17, 18). With the recent advancements within *in silico* prediction of BGCs and the increasing volume of sequenced rumen microbial genomes, increasing evidence of their presence in the rumen microbiome has been highlighted (17, 19, 20).

The Hungate1000 culture collection is a curated set of 410 cultured rumen microbial isolates established to represent the phylogenetic and functional diversity of the rumen microbiome. The collection comprises bacterial and archaeal strains isolated from ruminant hosts, spanning the dominant rumen-associated phyla. The genomes are high-quality (average 99.27% completeness) draft assemblies with an average genome size of 3,243,952 bp and a median assembly contiguity of 36 scaffolds (19). It provides a foundational genomic resource for studying rumen microbial ecology, metabolism, and host-microbe interactions, and has been widely used as a reference framework for rumen microbiome research. The current study aimed to mine the Hungate1000 culture collection to evaluate the bacteriocin biosynthetic potential of the catalogue to gain further insights into the mechanisms of microbial competition and decipher methods of bacterial communication in the rumen microbiome. This study expands on previous bacteriocin mining studies of the rumen microbiome by mining an increased number of genomes, using updated *in silico* prediction tools, and exploring the BGCs encoding putative novel bacteriocins. The study also highlights that bacteriocins mined from the Hungate1000 culture collection are found in the microbiomes of other ruminants and the human gut microbiome.

## RESULTS

### Abundance and phylogenetic distribution of BGCs within the Hungate1000

**Fig 1:**
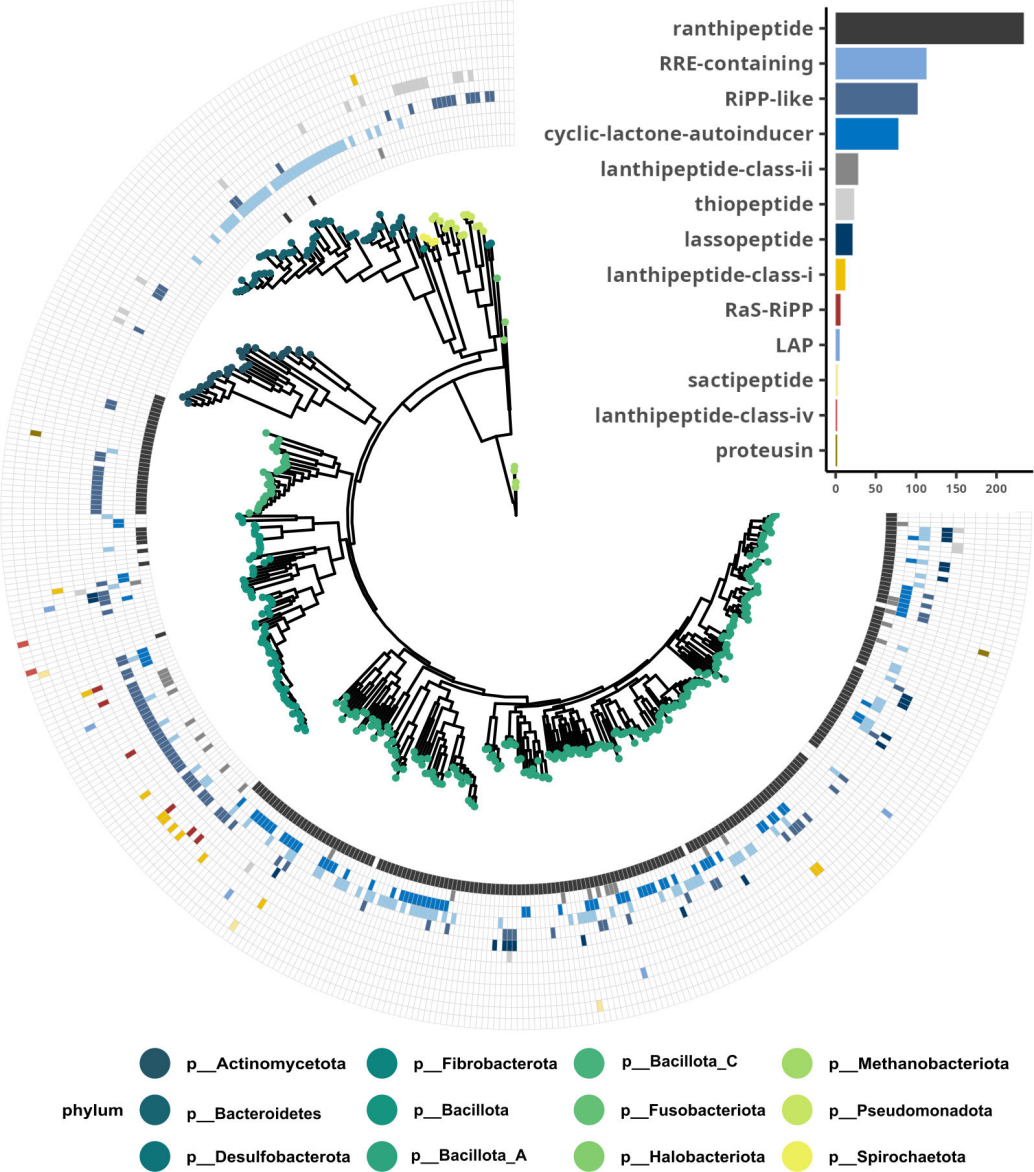
Phylogenetic distribution of RiPP/bacteriocin biosynthetic gene clusters within the Hungate1000 culture collection. The phylogenetic tree was constructed with 410 genomes using PhyloPhlAn v3 and is rooted to *M. wolinii* SH, an archaeal strain from the Hungate1000 collection. The colored tip points represent the phyla of each strain in the collection. The outer rings represent putative BGCs predicted by AntiSMASH. Inlaid in the corner of the plot is the total number of each class of BGC from each isolate. Bacteriocin BGCs are broadly distributed across the phylogeny but are primarily within the phylum Bacillota (Firmicutes). In particular, ranthipeptides dominate within this lineage, suggesting that members of Bacillota represent a major reservoir of ribosomally-synthesized antimicrobial potential within the rumen microbiome. The distribution pattern indicates that core peptides are phylogenetically structured rather than randomly dispersed across taxa.

**Fig 2:**
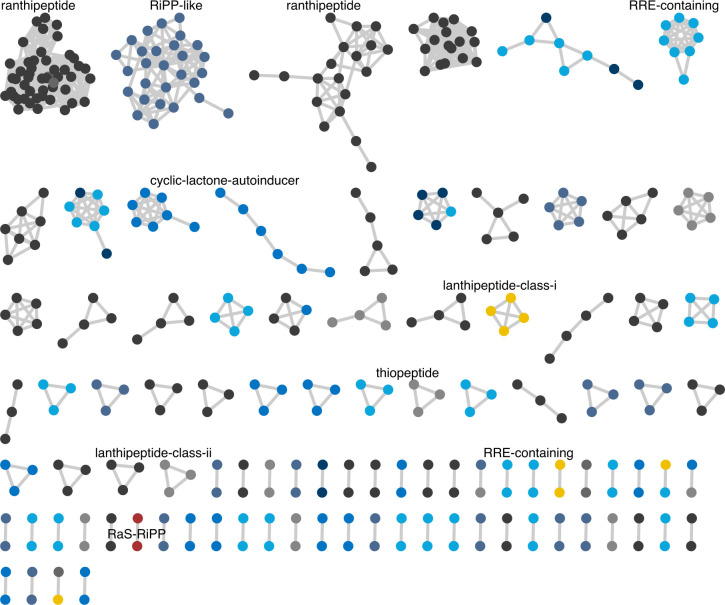
BGC similarity network created using BIG-SCAPE for RiPP BGCs. A BGC similarity network was generated using BIG-SCAPE based on pairwise domain architecture and sequence similarity among RiPP-associated clusters. The network was visualized in Cytoscape with each node representing a distinct BGC and colored according to its predicted class as assigned by antiSMASH. Edges connect clusters exceeding the similarity threshold (0.5) defined by BIG-SCAPE. Singleton clusters were excluded from network construction. Nodes are arranged by descending cluster size to emphasize major BGC families. The network reveals clear within-class clustering, indicating that BGCs with similar biosynthetic architectures (Pfam domains) group together into discrete families. Large, highly connected clusters are predominantly composed of ranthipeptides and RiPP-like BGCs, reflecting their expansion within the data set. In contrast, smaller and more fragmented clusters correspond to less prevalent bacteriocin classes, highlighting both conserved families and rarer, potentially unique biosynthetic lineages within the rumen microbiome.

The Hungate1000 culture collection contained 410 draft genomes of rumen-representative strains. All draft genomic data from the rumen isolates were downloaded from the Joint Genome Institute (JGI). The genomes were analyzed using antiSMASH and a total of 1,322 products were predicted across 380 of the 410 genomes within the collection (92.7%). AntiSMASH identified 750 putative RiPP/bacteriocin BGCs. Putative BGCs were selected for further analysis based on key modifying enzymes within the BGC. This was reduced to 476 high-quality BGCs by manually looking for core peptides within the BGC architecture (Table 1), of which 408 were deemed novel when the core peptide was not found in previous studies mining rumen isolates or the BAGEL4 database (17, 21). Here, novel bacteriocins are conservatively defined as core peptides lacking exact sequence matches to entries in BAGEL4 or previously reported rumen-associated bacteriocin data sets. This definition captures both entirely new peptides and sequence-divergent variants of known families, which can exhibit distinct activity spectra and functions, particularly given the strong sequence-function coupling in these small peptides. The majority of the BGCs identified belonged to the phylum Bacillota (Firmicutes), with the remainder distributed among Proteobacteria, Actinomycetota (Actinobacteriota), Spirochaetota, and Bacteroidota (Bacteroidetes) (Fig. 1; Fig. S1). Ranthipeptides (*n* = 254) were the most frequently detected putative BGCs and were found predominantly within the GTDB-defined phyla Bacillota_A and Bacillota_C, while being largely absent from Bacteroidota (*n* = 2), Actinomycetota (*n* = 0), and Bacillota *sensu stricto* (*n* = 1), consistent with clade-specific retention of this BGC. BGCs identified through the RiPP Recognition Element (RRE; a conserved domain that binds and positions RiPP precursor peptides for modification) were the second most abundant BGCs (*n* = 153) followed by RiPP-like peptides (*n* = 124) (Fig. 2). The RiPP-like subgroup encompasses both unmodified and modified ribosomally-synthesized peptides. This includes unmodified Class II bacteriocins, such as double-glycine leader-containing core peptides (e.g., Bacteriocin_IIc), and other non-modified bacteriocins such as lactococcin 972-like peptides (PF09683). This group also includes modified bacteriocins, including circular bacteriocins (e.g., BacteriocinIIc_cy and TIGR03651) and thiazole-containing bacteriocins identified by TIGR03693.

### Class I bacteriocins within the Hungate1000 culture collection

#### Circular bacteriocins

**Fig 3:**
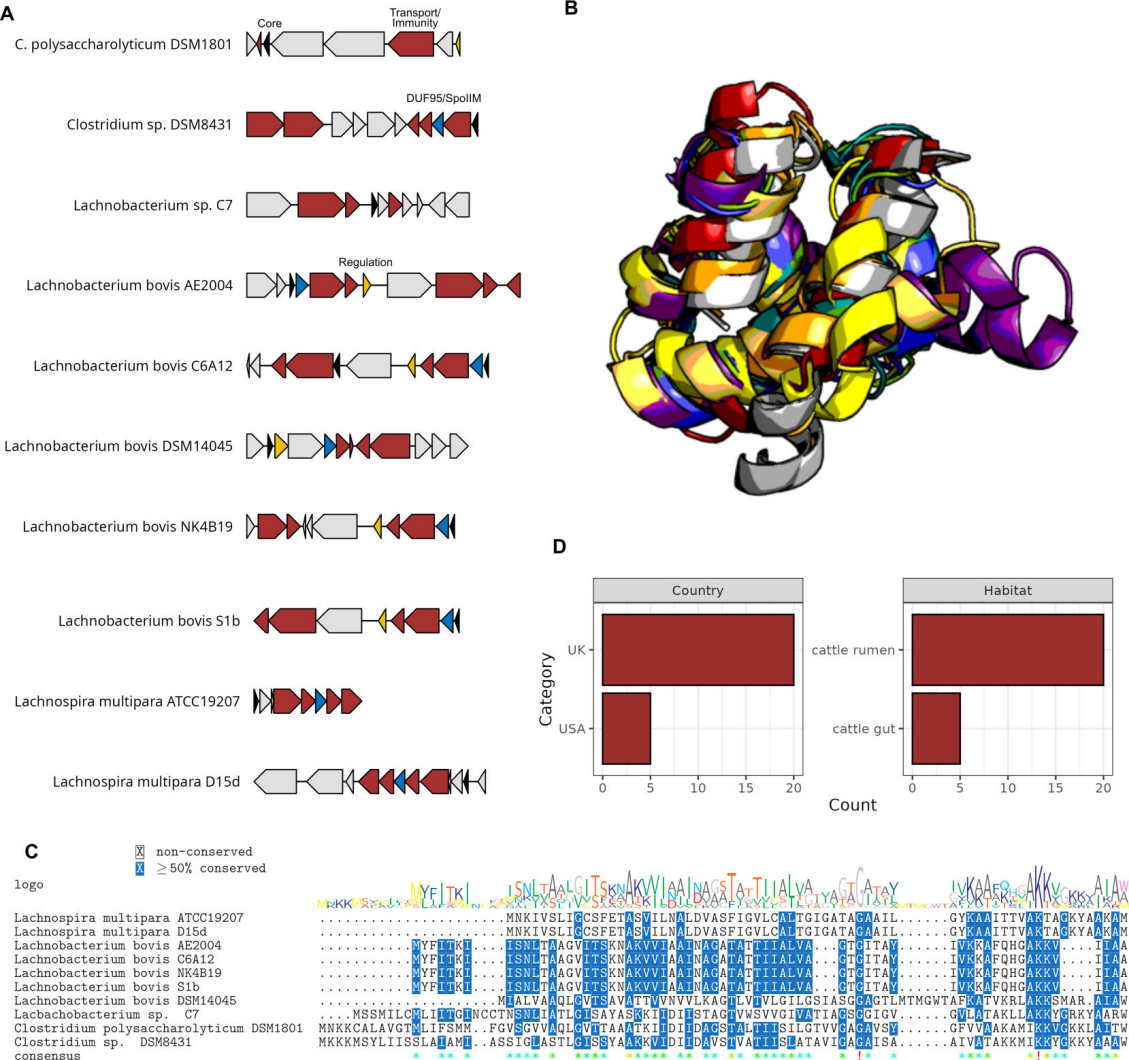
Circular bacteriocins in the Hungate1000 culture collection. (**A**) Biosynthetic gene clusters of putative circular bacteriocins. All strains are species without described production of circular bacteriocins to date. Black arrows represent the core peptide; red indicates proteins involved in transport and immunity; yellow denotes regulatory genes associated with the BGC; and blue represents the Stage II sporulation M protein (DUF95 family). The Stage II sporulation M protein is considered a class-defining protein family characteristic of circular bacteriocins. (**B**) Multiple sequence alignment of circular bacteriocin core peptides highlights shared core peptides between *Lachnobacterium bovis* strains. Although these isolates are from both cow and sheep ruminants, they were all isolated from New Zealand. (**C**) Predicted structure of circular bacteriocins using AlphaFold2 highlights multi-helical structures. (**D**) Circular bacteriocin core peptides identified from the Hungate1000 were detected exclusively in bovine-associated metagenomic samples. However, isolates encoding these peptides have also been recovered from other ruminant hosts, including sheep (WP_092479196.1).

Predicted circular bacteriocins mapped to BacteriocIIc_cy (PF12173.1). Circular bacteriocins have a head-to-tail cyclical structure from an N- to C-terminal covalent bond, and their production and export require a class-defining gene encoding a SpoIIM protein (PF01944, DUF95 protein family). Ten novel circular bacteriocins were predicted, all of which have predicted globular tertiary structures (Fig. 3A through C). For confidence scores of each protein structure prediction, see Fig. S2. Nine of these BGCs were predicted in *Lachnospiraceae*, a family without described production of circular bacteriocins to date. *Lachnobacterium bovis* DSM14045 encodes a peptide with 75.7% amino acid identity to Circularin A (WP_022748856.1). Multiple sequence alignment of circular bacteriocin core peptides highlights shared core peptides between four *Lachnobacterium bovis* strains AE2004 (cow rumen, New Zealand), C6A12 (cow rumen, NZ), NK4B19 (sheep rumen, NZ), and S1b (sheep rumen, NZ). This distinct core peptide was also detected in four cow rumen metagenomic samples from the United Kingdom and the United States, as well as in one cow fecal metagenomic sample from the United Kingdom, suggesting a broader geographic distribution of this BGC outside of New Zealand. *Clostridium polysaccharolyticum* DSM1801 was isolated from a sheep rumen from South Africa and encodes a novel circular bacteriocin with 63% identity to Amylocyclicin (WP_092479196.1), which could not be detected in metagenomic samples. All of these peptides are uncharacterized bacteriocins which have not been described previously by Azevedo *et al*. (17). Interestingly, these core peptides were found solely in cattle gut and cattle rumen metagenomic samples from studies in the UK and USA, suggesting host- or species-restricted occurrence of the peptides (Fig. 3D).

#### Lanthipeptides

**Fig 4:**
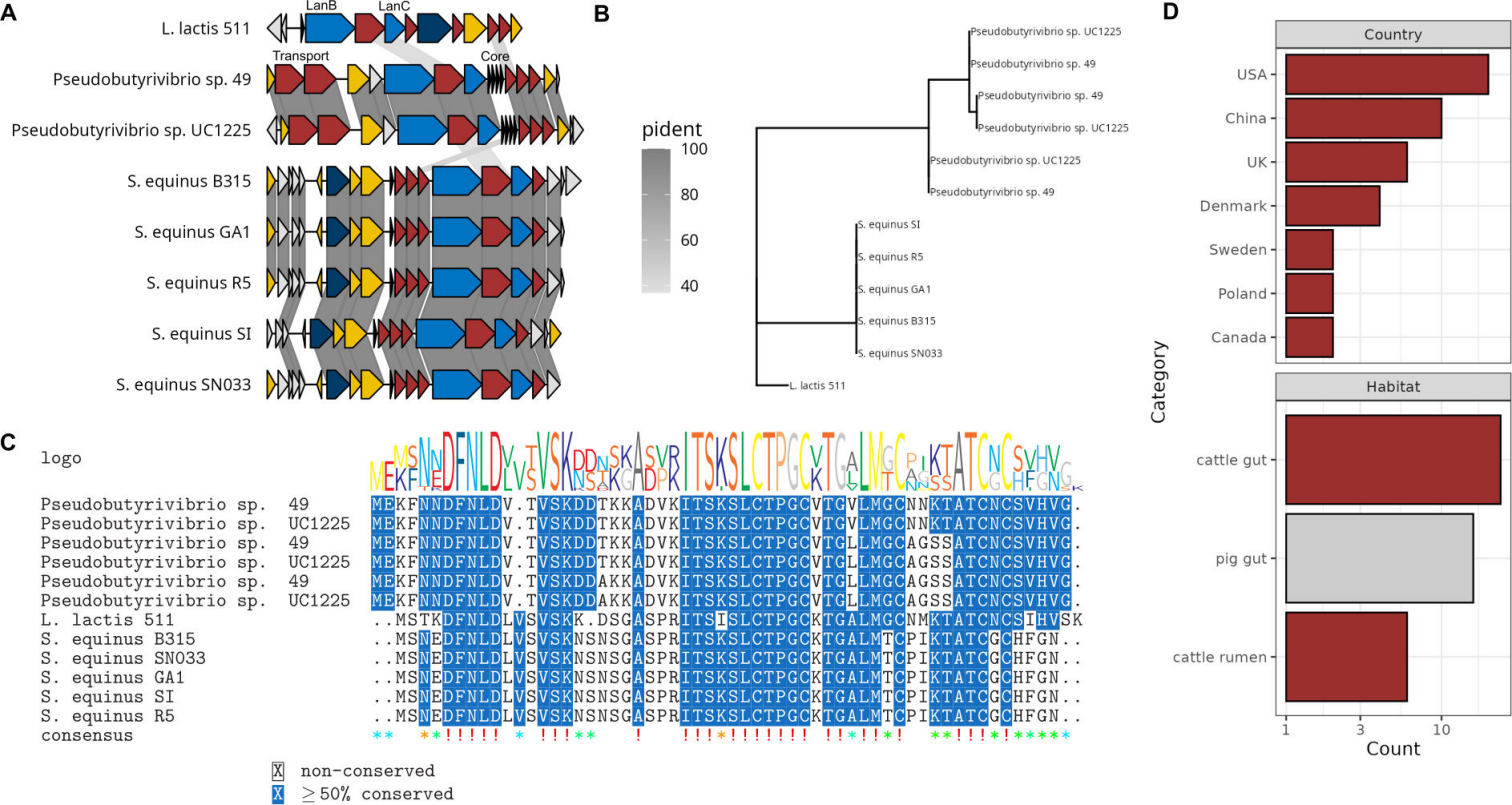
Nisin-like BGCs in the Hungate1000 culture collection. (**A**) Nisin-like biosynthetic gene clusters share a high degree of synteny within species-level boundaries. Blue open reading frames represent LanB and LanC proteins, dark blue represents proteases, and yellow represents regulation elements similar to NisR and NisK. A low percentage of amino acid identity (<40%) is shared between *Pseudobutyrivibrio* sp., *Streptococcus* sp., and *Lactococcus lactis* 511. (**B**) A phylogenetic tree of the nisin-like prepropeptides found in this study. (**C**) Multiple sequence alignment of the prepropeptides identified in this study. (**D**) Nisin-like core peptides encoded in *Pseudobutyrivibrio* sp. can be found in cattle gut and rumen metagenomes across seven countries.

AntiSMASH identified 42 lanthipeptide BGCs across 39 genomes, including *Streptococcus*, *Pseudobutyrivibrio,* and *Clostridium*. Of these, 11 are Class I, 30 are Class II, and 1 is Class IV. Type I lanthipeptides were further grouped based on the structure of the core peptide where subgroup I contains peptides structurally similar to nisin A, and subgroup II contains core peptides deemed “other.” Nisin E was identified across five separate *S. equinus* strains that demonstrate antimicrobial activity against *Clostridioides difficile* DPC6534 and *Clostridioides sporogenes* LMG10143 (22). These operons are >10 kb in length and contain genes encoding peptidases, LanB, LanC, ABC transporters, histidine kinase, and two-component regulator, suggesting a functional biosynthetic operon (Fig. 4A). *Pseudobutyrivibrio* strains were found to encode the same four putative core peptides with conserved “FNLD” and “CTPGC” leader and lipid II-binding motifs, respectively (WP_090169698.1, WP_090169700.1, WP_090169702.1, and WP_090169704.1) (Fig. 4B and C) (23). However, these share a markedly different cut site to the canonical nisin “GASPR” leader cleavage site observed in nisin variants originating from Lactobacillales and nisin O from *Blautia obeum* A2-162, suggesting lineage-specific proteases involved in the processing of the mature core peptide (24). Both *Pseudobutyrivibrio* strains were isolated from bovine ruminants located in the USA. Mapping of the *Pseudobutyrivibrio* nisin variants to the Global Microbial smORFs Catalogue (GMSC) identified these core peptides at 100% amino acid identity in cattle rumen/gut and pig gut metagenomic samples across seven countries, including Sweden, the UK, Poland, Denmark, China, and the USA (Fig. 4D). Subgroup II includes non-nisin Class I lanthipeptides, which are also modified by LanB and LanC but have a separate primary sequence to nisin, such as lacking the lipid II binding motif “CTPGC.” *Staphylococcus epidermidis* AG42 encodes Pep5 with 100% amino acid identity. *Peptostreptococcus* sp. D1 has a single core peptide similar to the ericin A-type lanthipeptide (33% identity, WP_091994375.1) that could not be identified in any metagenomic samples (Fig. S3) (25–27).

**Fig 5:**
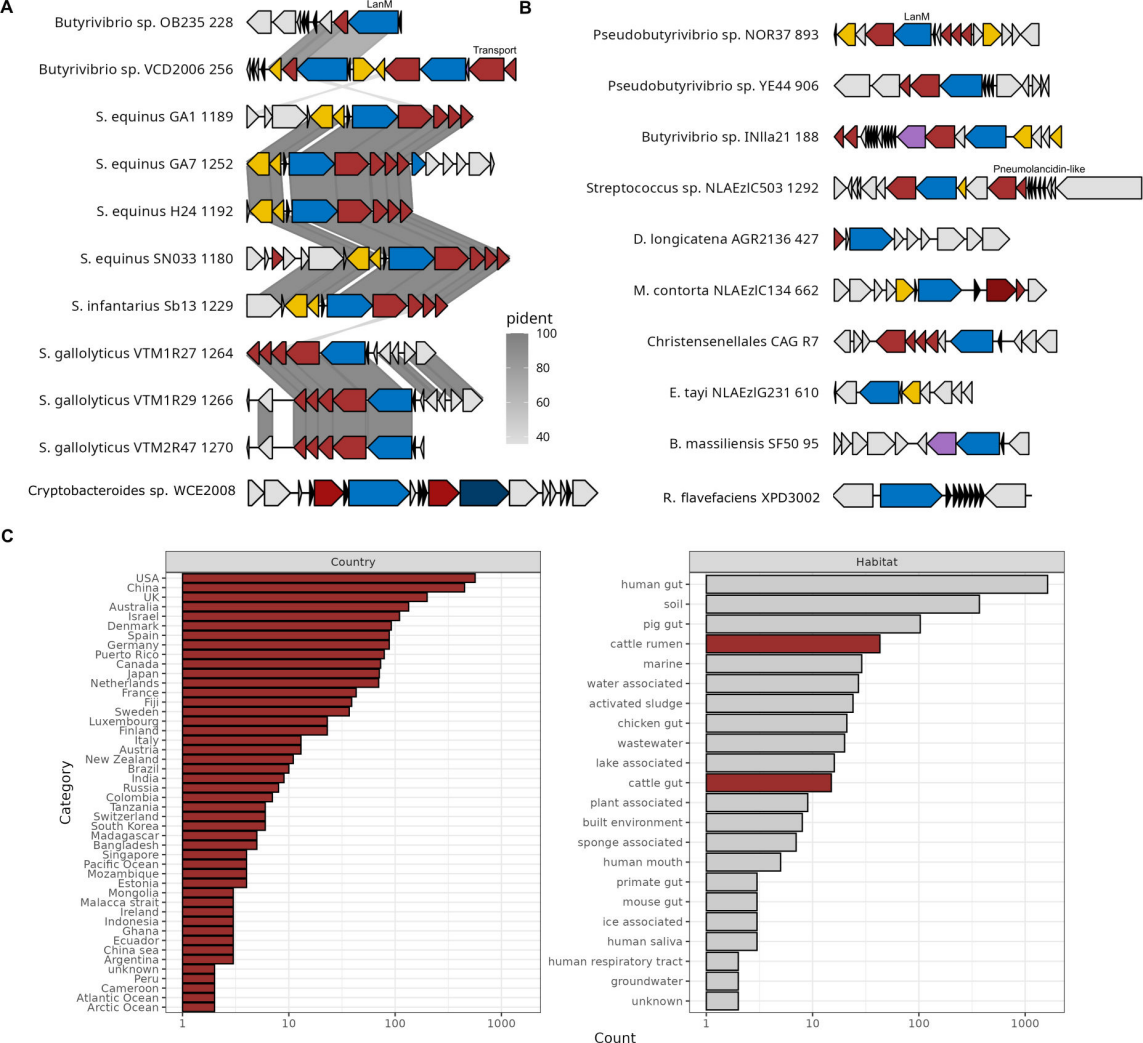
Operons containing Class II lanthipeptide BGCs in the Hungate1000 culture collection. (**A**) High degrees of synteny can be observed in the *Streptococcus bovis*/*equinus* complex (SBSEC). Blue represents genes encoding LanM proteins, purple represents genes encoding proteases, and yellow represents regulatory elements. Red are transport-associated genes. (**B**) Unique Class II lanthipeptide BGCs that lack synteny and are present within different genera. (**C**) Core peptides found within the Hungate are present in other microbiomes, including the human gut (*n* = 1,626), which is predominantly Pneumolancidin PldA2 and PldA1 core peptides (*n* = 1,490) encoded by *Streptococcus* sp*.* NLAEzlC503. Core peptides encoded by *Cryptobacteroides* sp. WCE2008 were identified predominantly in the cattle rumen microbiome (*n* = 40). *R. flavefaciens* XPD3002 encodes six distinct core peptides without homology to known Class II lanthipeptides, and these were detected in pig gut metagenomic samples.

**Fig 6:**
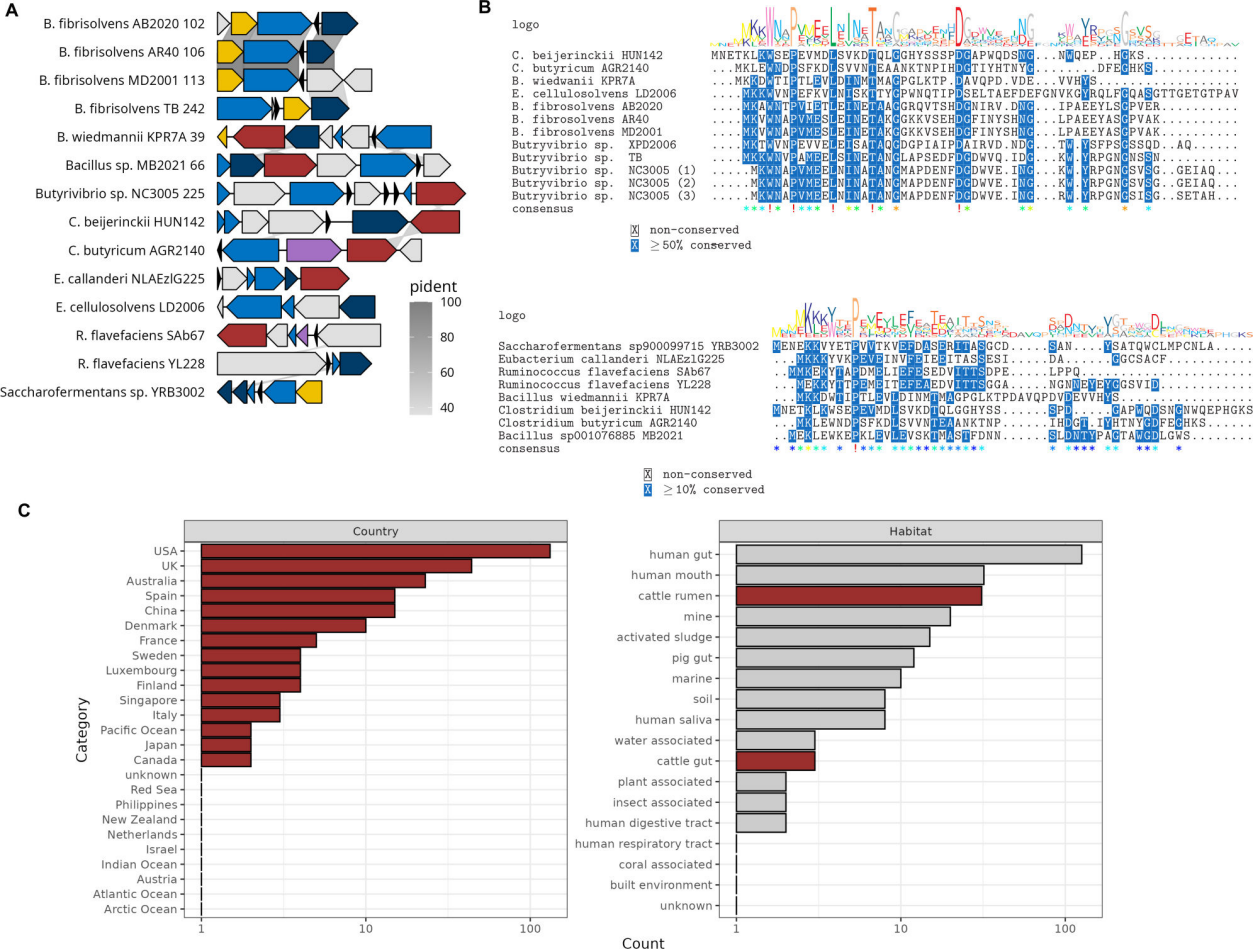
Lassopeptide BGCs predicted in the Hungate1000 culture collection. (**A**) Synteny analysis of lassopeptide biosynthetic gene clusters (BGCs), illustrating the conserved operon organization across representative genomes. Black arrows denote the core peptide gene. Yellow indicates regulatory proteins associated with BGC expression. Blue represents either an asparagine synthase-related protein or the canonical lasso peptide biosynthesis B2 protein, while dark blue denotes a nucleotidyltransferase family protein. Red arrows indicate transport-associated proteins, and purple highlights peptidase domain–containing proteins possibly involved in precursor processing. (**B**) Multiple sequence alignment of predicted lassopeptide core peptide sequences, highlighting conserved motifs and sequence features characteristic of this peptide class. (**C**) Distribution of lassopeptide-associated core peptides across host-associated metagenomes, demonstrating detection of this class in both human gut and cow rumen samples.

Class II lanthipeptides were the most abundant lanthipeptide class predicted and found mainly in *Bacilli* and *Clostridia* (Fig. 5A and B). Type 2 lantibiotic biosynthesis protein LanM (IPR017146) is a class-defining enzyme, which contains a dehydratase and cyclase domain in a singular protein; 30 putative Class II lanthipeptide BGCs were identified with heterogeneous putative core peptides (Fig. S6). *S. gallolyticus* VTM1R29, *S. gallolyticus* VTM1R27, and *S. gallolyticus* VTM2R47 were isolated from moose rumen samples and were found to share similar core peptides to Mutacin II (85.2% identity) from *Streptococcus* mutans (WP_074596227.1). Nukacin-like peptides were identified within *S. equinus* SN033, *S. equinus* GA-1, *S. equinus* GA-7, *S. equinus* H24, and *S. equinus* Sb13 (WP_303189437.1). These were isolated from deer rumen from New Zealand (NZ), cow rumen NZ, calf rumen USA, and cow rumen Australia, respectively. Each core peptide contains a double G-G or G-A peptide cleavage motif, a highly conserved “FTCC” C-terminal end of the peptide, and a “TVSx[ED]C” motif conserved among the majority of predicted Class II lanthipeptides (23). *Ruminococcus flavefaciens* XPD3002 contains six putative core peptides without matches to characterized Class II lanthipeptides (Fig. 6B). Core peptides from *Eubacterium ruminantium* HUN269 represent novel sequences from an order, Eubacteriales, without described lanthipeptide production to date. The peptides have 57.1%–75.9% amino acid identity to the lacticin 3147 A2 (LtnA2) mature core peptide, and each possesses a highly conserved “CPTxxCxxxC” motif, where x denotes any amino acid. These core peptides (WP_078787334.1, WP_078787335.1, and WP_078787348.1) were identified in metagenomic samples, with a single core peptide (WP_078787335.1) identified in 60 pig and chicken gut samples from multiple countries, including Australia (*n* = 39) and the UK (*n* = 18), suggesting dissemination of the BGC beyond NZ.

Four core peptides from *Streptococcus* sp. NLAE-zl-C503 share amino acid sequence identity with Pneumolancidins from *Streptococcus pneumoniae* (Fig. 6B). A single core peptide is identical to Pneumolancidin PldA2. This lantibiotic has proteolytic resistance to trypsin and pepsin and sensitivity to alpha-chymotrypsin (28). Notably, two of these core peptides were among the most frequently detected lanthipeptides across all metagenomic data sets, with the majority of detections originating from the human gut microbiome (*n* = 639 and *n* = 629; WP_016397469.1 and WP_016397470.1, respectively). The cross-host detection of pneumolancidin-associated peptides in both rumen-derived strains and human-associated microbiomes underscores their potential relevance within a One Health context. In total, 23 distinct Class II lanthipeptides could be identified in 2,207 metagenomic samples across 23 different biomes spanning 54 countries, with most of human gut origin (*n* = 1,626). Class II lanthipeptides could be found in 43 and 15 cattle rumen and cattle gut samples, respectively.

#### Lassopeptide

**Fig 7:**
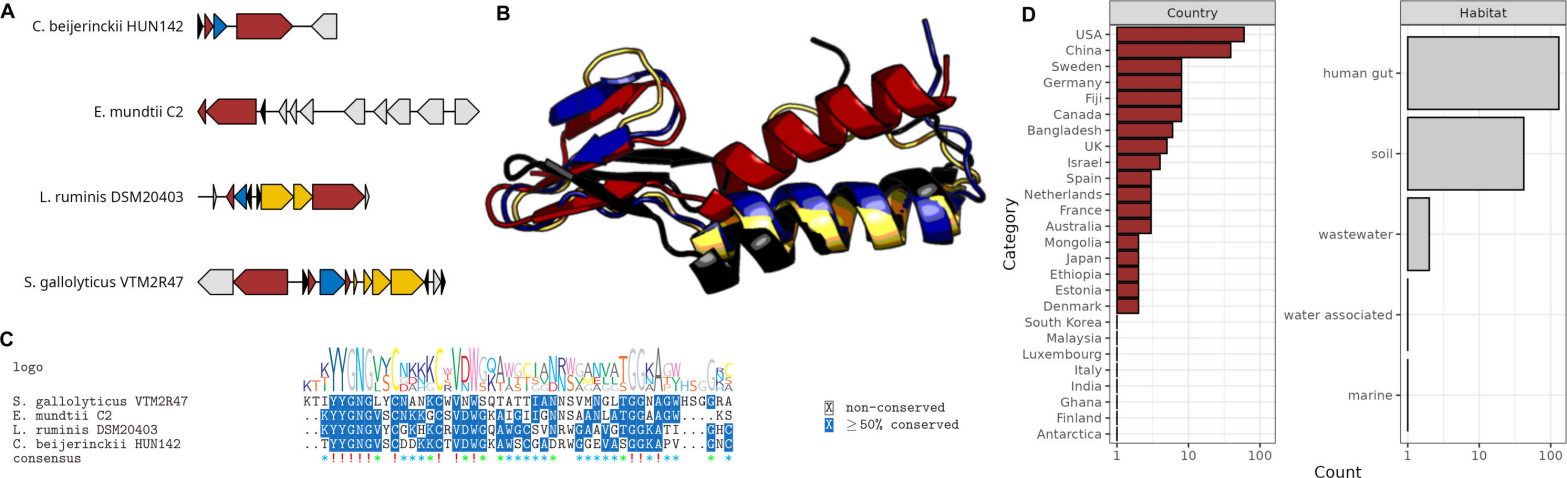
Pediocin-like bacteriocins in the Hungate1000 culture collection. (**A**) BGCs of the genomes encoding pediocin-like bacteriocins. Black indicates a core peptide, red is a transport/immunity protein, yellow colors are regulation proteins, and blue is a PedC/BrcD family bacteriocin maturation disulfide isomerase. (**B**) Multiple sequence alignment of the prepropeptides. (**C**) Structure of core peptides as predicted by AlphaFold. Structures share the same tertiary structure as pediocin PA-1, the class-defining peptide for Class IIa bacteriocins. (**D**) Pediocin-like bacteriocins were primarily found in the human gut from the USA with the core peptide from *Ligilactobacillus ruminis* DSM20403 (WP_014074090.1) found in the human gut and soil. This core peptide was identified in 176 samples ranging from human gut, soil, and the ocean.

Lassopeptides have an unusual loop-like structure that confers peptidase resistance and increased heat stability (29). These peptides have antimicrobial activity against both Gram-positive and Gram-negative bacteria (30). A recent *in silico* screen expanded the lassopeptide landscape and characterized novel lassopeptides with activity against clinically relevant vancomycin-resistant *Enterococcus faecium*, *Bacillus anthracis,* and *Mycobacterium smegmatis* (29). The typical lassopeptide BGC consists of an ATP-dependent cysteine protease with homology to transglutaminase (Transglut_core3; PF13471), and an ATP-dependent macrolactam synthetase with homology to asparagine synthetase (Asn_synthase; PF00733) (Fig. 6A) (31). Twenty-one potential lassopeptide BGCs were predicted, with the majority predicted among *Butyrivibrio* species. Twelve core peptides have a conserved “[GA]xxxxxxxD” motif, where x denotes any amino acid (Fig. 6B). This is characteristic of paeninodin and mesonodin lassopeptides along with a variable C-terminal region (29). However, little is known of their antimicrobial activity and spectrum of inhibition. The lassopeptide from *Clostridium butyricum* AGR2140 (WP_002580678.1), isolated from calf feces from NZ, was identified in 125 human gut and 32 human mouth samples, respectively, with the majority of gut samples originating from the USA. This core peptide was not identified in any ruminant animal metagenomes. Lassopeptides within the Hungate1000 culture collection could be found in 23 cattle rumen samples (Fig. 7C). Lassopeptide BGCs have been identified in *Butyrivibrio* sp. and have evidence of transcriptional activity in ruminants, suggesting that they are present and functional gene-products within the rumen (21).

### Class II bacteriocins within the Hungate1000 culture collection

AntiSMASH predicted four putative pediocin-like bacteriocin BGCs (Fig. 7A). All core peptides share the prototypical alpha-helix beta-sheet secondary structure (Fig. 7B) and a pediocin box motif “YYGNG” (Fig. 7C). *Clostridium beijerinckii* HUN142 has a novel core peptide (WP_026888094.1) similar to plantaricin C19 and sakacin G-type bacteriocins, a dedicated immunity protein (PF08951, IPR015046), C39 peptidase (TIGR01193, IPR005897), and a bacteriocin transport accessory protein (IPR005985). Notably, this BGC is also encoded upstream of an autonomous mobile genetic element of the transposase mutator family (PF00872, IPR001207), suggesting the gene cluster has been acquired via horizontal gene transfer. *E. mundtii* C2 encodes Mundticin ATO6 (WHL35506.1) and is the only peptide predicted to be localized on a plasmid. The core peptide from *Ligilactobacillus ruminis* DSM20403 was identified in human gut metagenomic samples (*n* = 176) (Fig. 7D).

Twenty-one unmodified two-component bacteriocin BGCs were predicted based on the presence of two successive core peptides within the bacteriocin BGC. A highly conserved double-glycine motif was observed for the majority of peptides, which signifies a putative cleavage recognition site. All predicted core-peptides contained one of AxxxA, GxxxG, and SxxxS motifs that give flexibility to the peptide and are characteristic of known unmodified two-component bacteriocins. Within the *Streptococcus bovis/Streptococcus equinus* complex (SBSEC) subset, regions of high synteny were observed between operons (Fig. S4). The SBSEC comprises a group of epidemiologically-significant organisms that include lineages potentially capable of causing disease in humans, including *Streptococcus gallolyticus* subsp. *gallolyticus* associated with colorectal neoplasia and infective endocarditis (32). The two-component bacteriocin Plantaricin EF (QGV13665.1, MGA3534237.1) encoded by *Lactiplantibacillus plantarum* AG30 was found in 25 cattle gut samples, but predominantly in human mouth (*n* = 98), human respiratory tract (*n* = 80), and human saliva metagenomic samples (*n* = 67).

### Prevalence of bacteriocins from the Hungate1000 in the global microbiome

**Fig 8:**
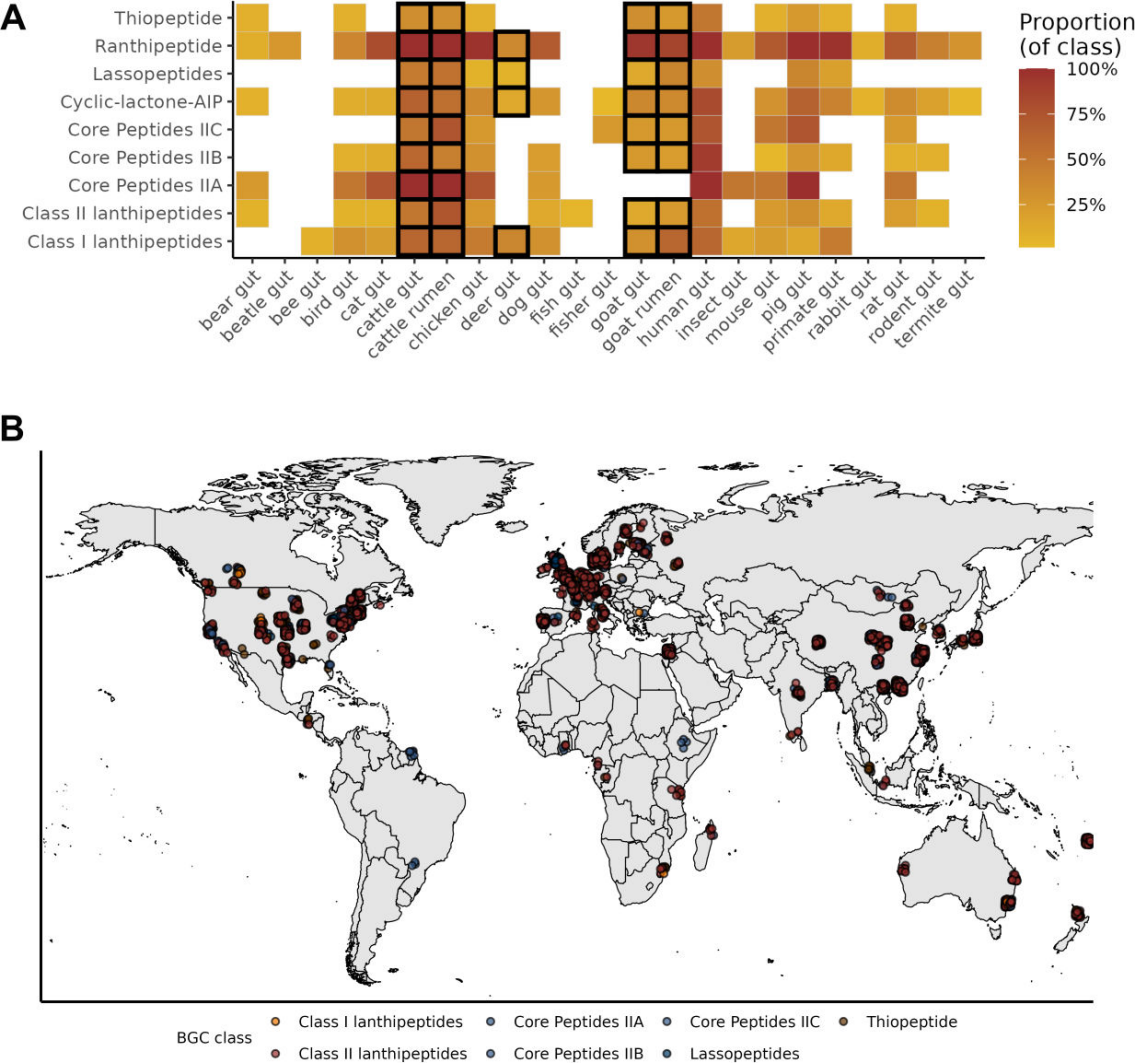
The bacteriocin core peptides identified in the Hungate1000 culture collection are found in ruminant animals and the human gut microbiome. (**A**) Bacteriocin core peptides found in the GIT of ruminant animals (highlighted in black) and human samples. The color is expressed as a percentage of the core peptides identified in this study that were detected in metagenomic samples. Samples are stratified by origin. (**B**) Bacteriocin core peptides are also globally disseminated in human gut and animal metagenomes. For clarity, the ranthipeptide was not included on the world map, as these are present in 32,512 samples and are ubiquitous to Clostridia.

Profiling of bacteriocin core peptides discovered in the Hungate1000 culture collection against the GMSC identified that the majority of core peptides, when detected, could be identified within cattle gut, cattle rumen, or human metagenomic samples (Fig. 8A). On average, distinct core peptides mined from Hungate1000 are found in between 2 and 6 other biomes, highlighting their dissemination beyond the rumen. However, when looking at the dispersal of the core peptides at the BGC class level, they are found between 2 and 23 other biomes, with the majority found in human and animal gut microbiome samples. Across the data set, ranthipeptides overwhelmingly dominate the BGC landscape, with 32,512 samples encoding a core peptide found in the Hungate1000. However, this class of BGC lacks well-characterized antimicrobial activity, and the sequences are widespread among Clostridia. Class II lanthipeptides (*n* = 2,343), followed by thiopeptides (*n* = 1,814), are the classes with the most prevalent core peptides with well-defined antimicrobial activity for each class. These peptides are also found globally disseminated (Fig. 8B). Class II lanthipeptides were found in 1,771 samples originating from the digestive tract of human and animal hosts. In contrast, unmodified or minimally modified bacteriocins, including unmodified two-component peptides IIB (*n* = 752) and pediocin-like (*n* = 176) are comparatively rare. These data demonstrate that bacteriocin core peptides identified in cultured rumen isolates are not confined to the rumen environment but are broadly disseminated across host-associated microbiomes, particularly within gastrointestinal ecosystems and beyond geographic boundaries.

## DISCUSSION

Recent advances in culturomics of the mammalian gut have highlighted the disparity between the ability to culture novel archaea and bacteria, particularly from the rumen, where less than 4% of operational taxonomic units (OTUs) have a representative isolate (19, 33, 34). The Hungate1000 catalogue represents every cultivated rumen-associated family consisting of 9 phyla, 48 families, and 82 genera (19). *In silico* screening uncovered 750 putative bacteriocin/RiPP BGCs, highlighting that the rumen microbiota has abundant biosynthetic potential and is an underexplored reservoir of potentially novel antimicrobial peptides. However, after manual curation for core peptides, this was reduced to 476 high-quality BGCs, of which 408 are novel. It is often cited that all bacteria produce at least one bacteriocin, but those actual predicted gene clusters encoding a core peptide are closer to 15%, a percentage seen in the human gut microbiome and rumen isolates (17, 18, 35, 36). Here, we report a rate of 30%, excluding the classes of bacteriocins that do not yet have extensively characterized antimicrobial activity, such as ranthipeptides and cyclic-lactone-autoinducer peptides. When these uncharacterized classes are included, the percentage of bacteriocin-positive isolates increases to 71.7%, a number closer to Klaenhammer’s suggestion of all bacteria producing at least one bacteriocin (35). Previous studies suggest a lower frequency of finding true positives as a result of the dependence on search tools to identify BGCs similar to existing BGCs, which is a limitation of *in silico* detection (17, 18).

The presence of bacteriocins in the rumen microbiome suggests that they likely play roles in structuring microbial interactions within this densely populated and metabolically interdependent community (12, 20, 37, 38). In this ecosystem, the antimicrobial activity of bacteriocins, together with their regulated induction, suggests that they may confer a selective advantage by mediating strain-level competition, facilitating stable colonization, and potentially contributing to signaling interactions such as quorum sensing, with downstream effects on populations involved in fiber degradation, fermentation, and methane production (17, 20, 37, 39). Lassopeptide-associated genes originating from *Lachnospira multipara* D15d and *Clostridium butyricum* ARG2140, both of which are hydrogen-producing members of the Hungate1000, were found to be transcriptionally active *in vivo* (21). Although this does not confirm peptide activity *in vivo*, the sustained expression of these loci suggests a functional role. As bacteriocin production is often regulated through quorum sensing and its expression is condition-dependent, these effects are likely spatially and temporally constrained within the rumen (3, 40).

Rumen metabolism is underpinned by tightly coupled syntrophic networks, with interspecies hydrogen transfer linking the activity of primary fermenters to that of hydrogenotrophic partners such as methanogens (41–43). Consequently, bacteriocin-mediated inhibition of selected hydrogen-producing taxa could exert community-wide effects disproportionate to their abundance by altering hydrogen flux, reshaping fermentation pathways, and modulating downstream methanogenesis (41–43). Selectively targeting hydrogen-producing populations through narrow-spectrum antimicrobials is therefore likely to alter hydrogen availability. Notably, lantibiotic-producing streptococci inhibit *Clostridium* species, with some species contributing substrates for CH₄ production (16). However, rumen methanogenic archaea are comparatively less diverse than bacteria, with *Methanobrevibacter* dominating hydrogenotrophic populations, and methane output is closely linked to microbial composition and the transcriptional activity of methanogenic pathways of Archaea (44). Although most rumen methanogens possess a pseudomurein cell wall that could represent a potential bacteriocin target, conclusive data remain elusive for most bacteriocin classes (45, 46). Nisin has been shown to inhibit *Methanobrevibacter boviskoreani* JH1 *in vitro* at 12 μM, highlighting that lanthipeptides are active against methanogenic Archaea (47). However, indirect ecological effects mediated through bacterial interactions likely represent the primary route by which bacteriocins influence methane production. This highlights the importance of considering potential off-target effects on rumen methane dynamics, target strain specificity, and ecological consequences when evaluating bacteriocins as microbiome-modulating agents (21, 48–50).

Given that lantibiotic and bacteriocin production is a common feature of many probiotic organisms, these traits may offer a route to rationally engineer rumen microbial ecology through the introduction of bacteriocin-producing strains (51). This raises the possibility of developing generally regarded as safe (GRAS) or qualified presumption of safety (QPS) strains to manipulate rumen function. While direct-fed microbials are known to improve feed efficiency, weight gain, and animal health, their effects on methane emissions remain poorly characterized in comparison (52, 53). *Lactobacillus buchneri* administered to silage inoculants achieved over 83% CH_4_ reduction when administered in combination with absorbents, such as wheat bran, highlighting the role that LAB can play in manipulating rumen function (54, 55). A recent silage study further strengthens this link to bacteriocin-producing LAB showing that two bacteriocin-producing *Lactiplantibacillus plantarum* strains, ATCC14917 and LP1-4, reduced *in vitro* ruminal methane production while improving digestibility (56).

Interestingly, the identification of nisin variants within the rumen-associated collection suggests that nisin-like antimicrobials may play roles in microbial competition beyond food-associated environments (6, 57). Four natural nisin variants were discovered from *Streptococcus*, *Lactococcus,* and *Pseudobutyrivibrio,* with nisin E produced by multiple *Streptococcus equinus* strains of rumen origin (22). Heterologous expression of *Pseudobutyrivibrio* variants has highlighted their potent activity against human gut anaerobes such as *Coprococcus comes* MSK.11.23 and *Dorea formicigenerans* MSK.17.61 (6, 57). Consistent with an ecological role beyond direct killing of susceptible species, oral nisin administration in pigs reversibly altered gut community composition decreasing Gram-positive taxa and reshaping metabolic function, including reduced acetate and butyrate pathways and increased propionate-associated pathways, with corresponding reductions in fecal short-chain fatty acids (58). The most relevant species-level decreases include *Anaerostipes* and *Dorea*, which the nisin study reported were reduced during treatment, alongside decreased acetate- and butyrate-associated pathways (58). An increase in *Prevotella* and *Phascolarctobacterium* following nisin treatment was also observed, together with enrichment of propionate-associated pathways. *Prevotella* is frequently associated with higher propionate and lower methane, while *Phascolarctobacterium* converts succinate to propionate, supporting metabolic hydrogen flux away from methanogenesis; this could be possible in the rumen if similar bacterial targets are susceptible (41–43, 59, 60). Class II lanthipeptides were the most abundant and globally prevalent lanthipeptide class in the global microbiome (*n* = 2,343). The predominance in gut-associated genomes and metagenomes suggests that these peptides are evolutionarily adapted to intestinal ecosystems. Notably, *Ruminococcus flavefaciens* FD-1 encodes multiple core peptides, several of which exhibit activity against the rumen bacterium *Ruminococcus albus* (41, 61). This further demonstrates that rumen-derived bacteriocins can inhibit other rumen-associated taxa suggesting that they may contribute to eco-evolutionary processes underlying microbial interactions in the rumen. Together, these findings indicate that lanthipeptides represent candidates for targeted manipulation of microbial communities including methane-associated processes within the rumen (60, 62).

Although relatively few Class II bacteriocins were identified in the Hungate1000, four pediocin-like peptides were predicted. This class has also been shown to reduce methane production *in vitro* (9). *Streptococcus gallolyticus* VTM2R47 encodes a peptide similar to ubericin A, originally described in *Streptococcus uberis,* which is a bovine-associated species linked to mastitis (63). Bacteriocins also act as signaling peptides, potentially facilitating interbacterial messaging and communication (quorum sensing) (64–67). This highlights the importance of considering broader ecological consequences, including the potential dissemination of pathogenic traits and species, and breaking inter-species microbial networks.

Consistent with these ecological roles, Hungate-associated bacteriocins are not geographically restricted but are more frequently detected in gut microbiomes (Fig. 8B). This widespread distribution suggests that rumen bacteriocin BGCs are maintained by selective pressures linked to gut ecological functions such as competition for nutrients by distinct gut-associated taxa. It is important to note that inclusion in this analysis required 100% amino acid identity between a detected bacteriocin core peptide and a sequence in the collection, intentionally excluding closely related variants and naturally diversified homologs. Also, the GMSC is restricted to assembled contigs, meaning that bacteriocin core peptides can only be detected with sufficiently high relative abundance for assembly. Therefore, the distributions reported here represent a conservative estimate of the true prevalence of these bacteriocins and their variants in the global microbiome. These findings highlight rumen bacteriocin BGCs as globally disseminated with potential implications across One Health domains, spanning animal health, the environment, and the broader dynamics of gut-associated antimicrobial ecology (Fig. 8A and B).

The *in silico* analysis of the Hungate1000 culture collection identified 408 putative novel bacteriocin BGCs across diverse rumen-associated genomes, highlighting the rumen as an underexplored reservoir of antimicrobial diversity. Although experimental validation of bacteriocin production will require substantial further work, scalable heterologous expression offers a feasible route to overcome limitations associated with native production and may accelerate functional characterization and application (68). More broadly, the Hungate1000 also represents a valuable resource for the rational design of synthetic rumen microbial consortia for introduction via silage inoculants. Incorporating bacteriocin-producing strains into such consortia may enable the reshaping of microbial interactions toward a low-methane phenotype by modulating competitive dynamics and metabolic cross-feeding networks, rather than relying solely on direct inhibition of methanogens or hydrogen-producing bacteria via bacteriocins (54, 69, 70). The frequency of putative bacteriocin production within ruminal strains was greater than 30%, doubling the frequency previously suggested in the mammalian gastrointestinal tract. These findings position the Hungate1000 as a valuable resource to interrogate and potentially modulate methane emissions *in vitro* and *in vivo*.

## MATERIALS AND METHODS

### Detection of BGCs

Draft genome sequences of 410 ruminal bacteria and archaea were accessed from the Joint Genome Institute in April 2020. The genomes were annotated using Bakta v1.9.1 with database v5.0 to have full annotation when manually curating BGCs (71). BGCs were identified using antiSMASH v7.0 on Bakta “gbff” files, with default settings (72). Both antiSMASH BGCs and whole genome annotation for all assemblies can be found at Zenodo (https://doi.org/10.5281/zenodo.18600355). BIGSCAPE v1.1.5 was used with the parameters “-c 8 --cutoffs 0.3 0.5 0.75 0.8 0.85 0.9 --clan_cutoff 0.3 0.9 --mode global --banned_classes PKSI PKSother NRPS Saccharides Terpene PKS-NRP_Hybrids” to categorize the BGC clusters (73). Here, BGCs were categorized based on the presence/absence of critical biosynthetic protein Pfams and antiSMASH. A network cutoff value of 0.5 was chosen for the network analysis for an appropriate resolution between BGCs. The networks were visualized in Cytoscape v3.10.1 using the “prefuse force directed layout” and colored by BGC class (74). Platon V1.7 was used to predict plasmids in the 410 genomes using the default database and “strict” mode (75). Strain identifiers and accessions were manually cross-referenced with other studies, which included a limited number of the strains in the Hungate1000, to determine the novelty of BGC prediction along with an extensive database of lanthipeptide core peptides (17, 21). BGC predictions were manually curated to keep BGCs with core peptides. Core peptides were searched against the BAGEL4 bacteriocin database using BLAST v2.12.0, and matches with a 100% hit on the core peptide were deemed “known” (See section: Prediction of core-peptides) (76–78). A table of known core peptides can be found in Table S4, Sheet 1, along with the studies which initially identified each BGC. In order for a BGC to be retained, each BGC required a putative core peptide that was localized near the BGC machinery (i.e., LanM for class II lanthipeptide) and contained class-specific motifs within the core peptide or a hit to a core peptide in the BAGEL4 database. If protein-coding sequences (CDS) below 100 AA were found in the same 20 kb neighborhood with no homology to an existing member of the class or did not include required class-specific motifs within the core peptide or the core peptide lacked proximity to anchoring BGC-related domains, they were not deemed high confidence BGCs. Short protein-coding sequences below 100 AA, encoding C, T, and S residues, were deemed putative lanthipeptides, as these are required for post-translational modification. The “CTPGC” motif was also used to search for nisin-like lanthipeptides specifically. Thiopeptides required multiple C, S, or T residues in the C-terminal end of the protein coding sequence below 100 AA or an annotation as “Thiocillin/thiostrepton family thiazolyl peptide” by Bakta. Pediocin-like peptides required a “YGNG” motif, and two-component unmodified bacteriocins required two proximal CDS below 100 AA that contain either a “AxxxA,” “GxxxG,” or “SxxxS” motif, where “x” denotes any amino acid. Circular bacteriocins required a Stage II sporulation protein M (PF01944, previously DUF95) encoded within the BGC along with CDS below 100 AA annotated as Bacteriocin_IIcy (core peptide). Lassopeptides required a core peptide below 100AA with a “Gx7D”, where x denotes any seven amino acids. Ranthipeptide and cyclic lactone autoinducers were identified by their respective AntiSMASH domains, identifying these short CDS.

### Phylogenetic distribution of BGCs

Taxonomy was assigned to each draft genome using the Genome Taxonomy Database Toolkit (GTDB-Tk) v1.4.0 average nucleotide identity (ANI) method using standard settings (79). A phylogenetic tree was constructed using PhyloPhlAn v3.0.60 on draft genome assemblies (80). PhyloPhlAn reconstructs phylogenies using hundreds of conserved, single-copy protein-coding genes shared across bacteria and archaea, making it suitable to include archaeal and bacterial genomes in a single tree (80). PhyloPhlAn was run with the parameters “--diversity high --fast” using diamond with the parameters “--threads 1 --outfmt 6 --more-sensitive --id 50 --max-hsps 35 -k 0” (77). The config file can be found at https://github.com/DEHourigan/hungate_1000. The tree was decorated and plotted using the ggtree v3.8.2 R package (81).

### Synteny

An all-vs.-all blast was performed with all protein-coding sequences within operons using BLASTp v2.12.0 (78). Operons were plotted using the gggenomes v0.9.9.9 R package, and gene functions were colored based on Bakta annotation (82).

### Prediction of core peptides

Putative core peptides were identified by the presence of key motifs with short open reading frames (sORFs) (<100 aa). Each sORF was also searched against the nr and BAGEL database using BLAST v2.12.0 (78). sORFs were then manually checked based on their sequence and location in the operon to determine if they are part of a putative bacteriocin BGC. Amino acid sequences were aligned using MUSCLE from the msa R package v1.32.0 and visualized using msaPrettyPrint (83, 84). Sequences were clustered per bacteriocin type using CD-HIT v4.8.1, with default settings when appropriate (85). For phylogenetic tree construction, sequences were aligned using MUSCLE v3.8.1551 and RAxML-NG v1.2.1 with 100 bootstrap replicates (83, 86). Trees were decorated using the ggtree v3.8.2 R package (81).

### Structure prediction

Core peptide structures were predicted for unmodified Class II bacteriocins using AlphaFold v2.3.2 using the settings “--model_preset = monomer --enable_gpu_relax = true” (87). Five structural models were generated per peptide, and the highest confidence model, based on AlphaFold ranking metrics, was selected for downstream visualization and analysis using the PyMOL Molecular Graphics System (v2.6; Schrödinger, LLC). All predicted structures and corresponding mature peptide sequences are publicly available at https://github.com/DEHourigan/hungate_1000. AlphaFold per-residue pLDDT datapoints were extracted using Alphapickle v1.4.0 (https://doi.org/10.5281/zenodo.5708708) and plotted using Python v3.9.5.

### Metagenomic mapping of bacteriocin core peptides from Hungate1000

Bacteriocin core peptides identified in the Hungate1000 culture collection after manual curation were mapped against the Global Microbial smORFs Catalogue v1.0.

Databases were downloaded and formatted using the utility tool as follows “gmsc-mapper downloaddb --all.” Searches were performed with “gmsc-mapper --aa-genes” against the databases clustered at 100% amino acid identity (termed 100 AA smORF catalogue). Only hits with 100% amino acid identity over the length of the subject were retained, thereby restricting downstream analyses to core peptides uniquely detected in the Hungate1000 culture collection. Sample metadata were downloaded from https://gmsc-api.big-data-biology.org/files/GMSC10.metadata.tsv. The bacteriocin core peptide heatmap and world map were plotted using ggplot2 with “map_data” and R v4.4.3. Longitude and latitude coordinates were taken from the GMSC metadata file when present.

## Data Availability

All 410 cultivated reference genomes are available from the Joint Genome Institute under the DOI https://doi.org/10.46936/10.25585/60000534 or from NCBI under the Umbrella project accession PRJNA471733. Scripts can be found at https://github.com/DEHourigan/hungate_1000. Data generated can be found at Zenodo (doi: https://doi.org/10.5281/zenodo.18600355).

## SUPPLEMENTAL MATERIAL

Supplemental Table Captions msystems.00195-26-s0001.docx Captions for Tables S1-S6.

Supplemental Figures msystems.00195-26-s0002.pdf Fig. S1 to S4.

Table S1 msystems.00195-26-s0003.csv GTDB-assigned taxa to isolates from the Hungate1000.

Table S2 msystems.00195-26-s0004.csv Antismash7 result overview from the Hungate1000.

Table S3 msystems.00195-26-s0005.xlsx BAGEL4 core peptide blast hits from the Hungate1000.

Table S4 msystems.00195-26-s0006.xlsx Core peptides from the Hungate1000.

Table S5 msystems.00195-26-s0007.xlsx BGCs on plasmids from the Hungate1000.

Table S6 msystems.00195-26-s0008.html HTML-formatted version .tsv table on Zenodo.

ASM does not own the copyrights to Supplemental Material that may be linked to, or accessed through, an article. The authors have granted ASM a non-exclusive, world-wide license to publish the Supplemental Material files. Please contact the corresponding author directly for reuse.
