# Supplementary material for: The Hungate1000 prokaryotic culture collection encodes a wide variety of bacteriocins
Source: mSystems. 2026 May 27;11(6):e00195-26. doi: 10.1128/msystems.00195-26 (PMC13289728; doi:10.1128/msystems.00195-26)
Supplement: Supplemental Table Captions — Captions for Tables S1-S6. [file msystems.00195-26-s0001.docx]

# *The Hungate 1000 prokaryotic culture collection encodes a wide variety of bacteriocins*

# Supplementary Table Legends

Supplementary Table S1. GTDB-assigned taxa to isolates from the Hungate1000.

Supplementary Table S2. Antismash7 result overview from the Hungate1000.

Supplementary Table S3. BAGEL4 core peptide blast hits from the Hungate1000.

Supplementary Table S4. Core peptides from the Hungate1000.

Supplementary Table S5. BGCs on plasmids from the Hungate1000.

Supplementary Table S6. This table is the HTML formatted version <8 Mb) of "Supplementary_table_6_GMSC.tsv" which can be found on Zenodo.
