# Supplementary material for: The Hungate1000 prokaryotic culture collection encodes a wide variety of bacteriocins
Source: mSystems. 2026 May 27;11(6):e00195-26. doi: 10.1128/msystems.00195-26 (PMC13289728; doi:10.1128/msystems.00195-26)
Supplement: Supplemental Figures — Fig. S1 to S4. [file msystems.00195-26-s0002.pdf]

The Hungate1000 prokaryotic culture collection encodes a wide variety of bacteriocins

Supplementary information

David Hourigan,[a,b] Lorraine Draper,[a,b] Sinead C Leahy,[d,e] Graeme T Attwood,[d] William J Kelly,[d] Catherine Stanon,[a,c] Colin Hill,[a,b] Paul Ross[a,b,c]#

[a] APC Microbiome Ireland, Biosciences Institute, Biosciences Research Institute, College Rd, University College, Cork, Ireland

[b] School of Microbiology, University College Cork, College Rd, University College, Cork, Ireland

[c] Teagasc Food Research Centre, Moorepark, Moorepark West, Fermoy, Co. Cork, Ireland

[d] AgResearch Ltd., Grasslands Research Centre, Palmerston North, New Zealand

[e] New Zealand Agricultural Greenhouse Gas Research Centre (NZAGRC), Palmerston North, New Zealand

#Address correspondence to p.ross@ucc.ie

Author order was determined on the basis of seniority.

Running Head: Identification of bacteriocins in the Hungate1000

(a)

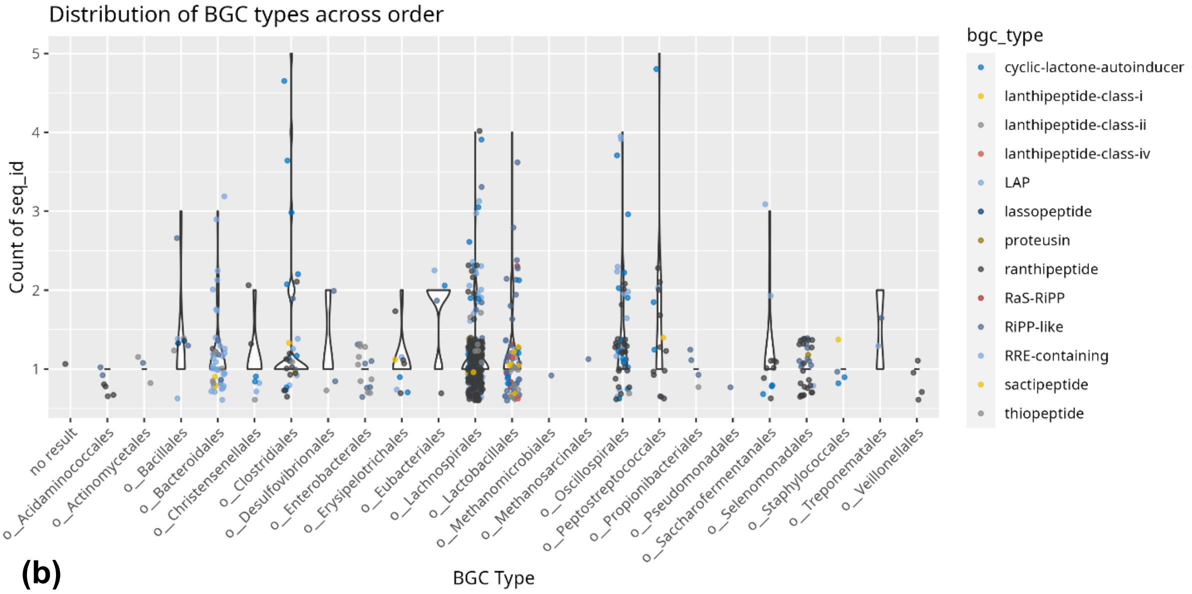

(b)

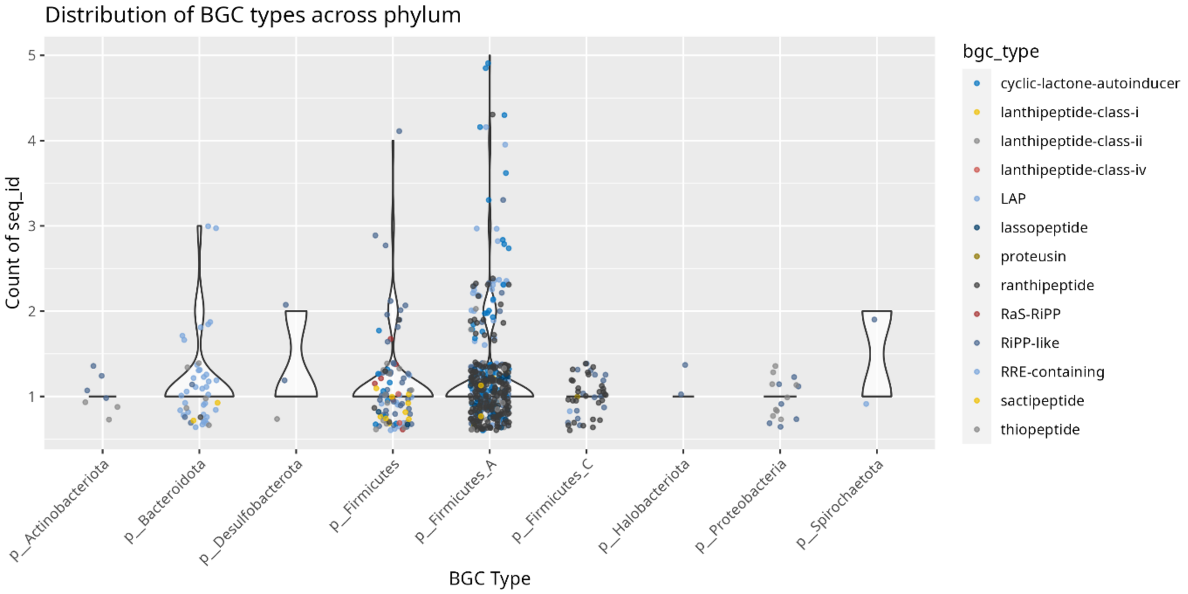

Figure S1. Table of the distribution of biosynthetic gene clusters predicted by antismash7 stratified by **(a)** order, **(b)** phylum and BGC class.

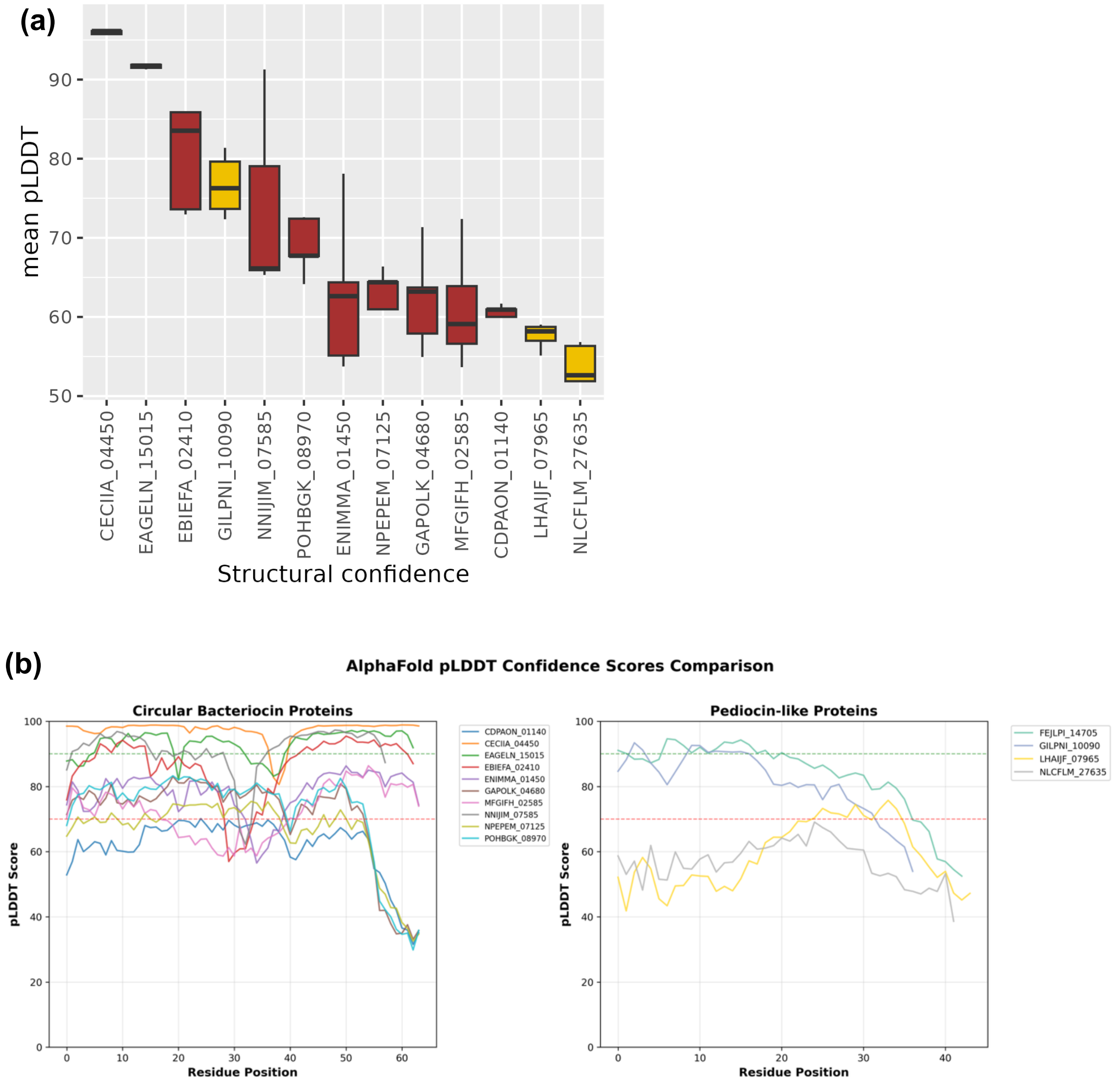

Figure S2. Alphafold predicted confidences for predicted structures of class IIa and class IIc bacteriocins. (a) Mean pLDDT scores for each of the five AlphaFold-predicted models per bacteriocin. Circular bacteriocins are shown in red, while Class IIa (pediocin-like) bacteriocins are shown in yellow. **(b)** Per-residue pLDDT profiles for each predicted core peptide, illustrating positional variation in model confidence across the peptide sequence. These scores reflect prediction confidence and do not constitute experimental validation.

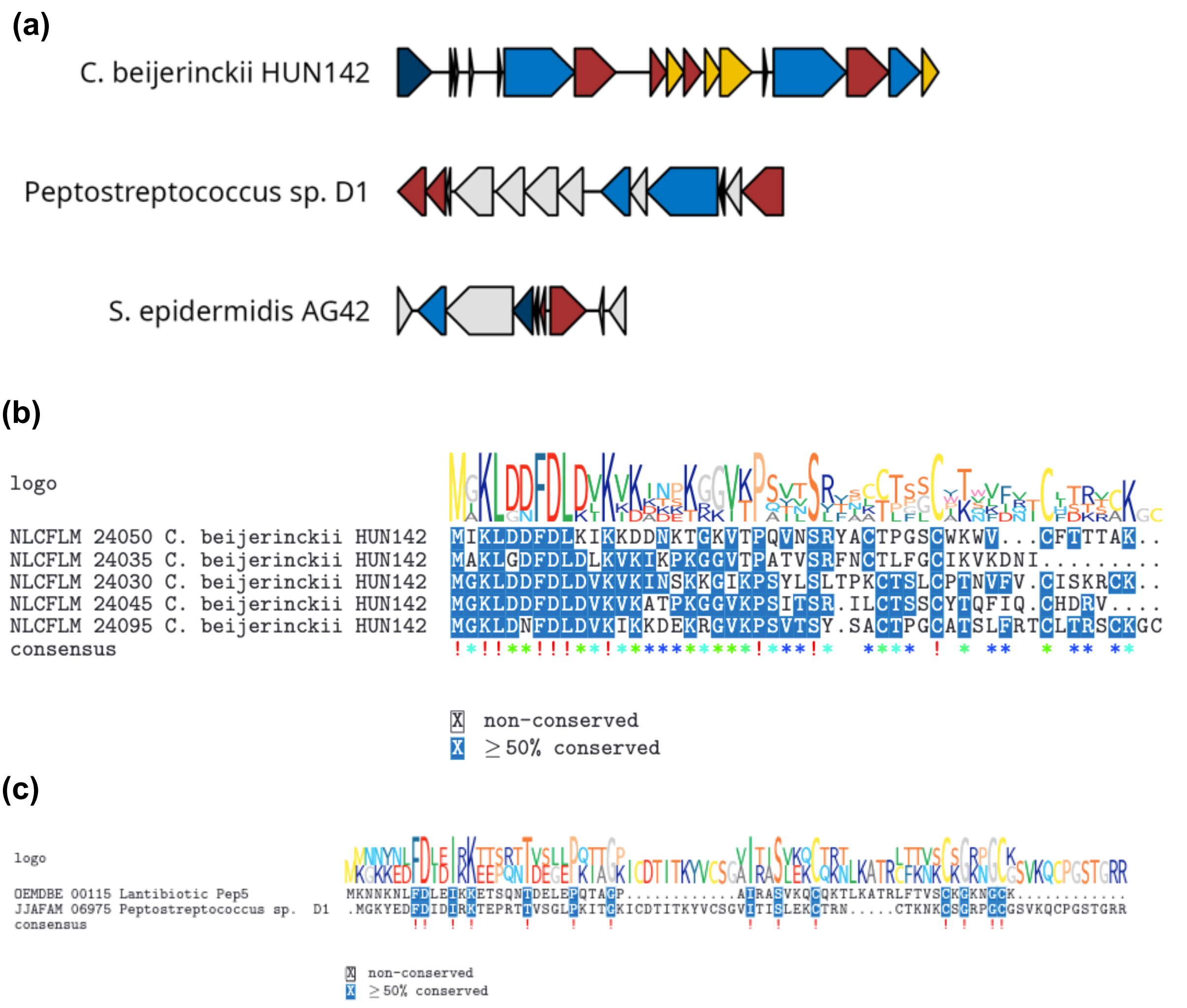

Figure S3. Non-nisin class I lanthipeptide core peptides. (a) Operons for genomes encoding non-nisin like class I lnthipeptide BGCs in the Hungate1000. **(b)** Core peptides from these BGCs.

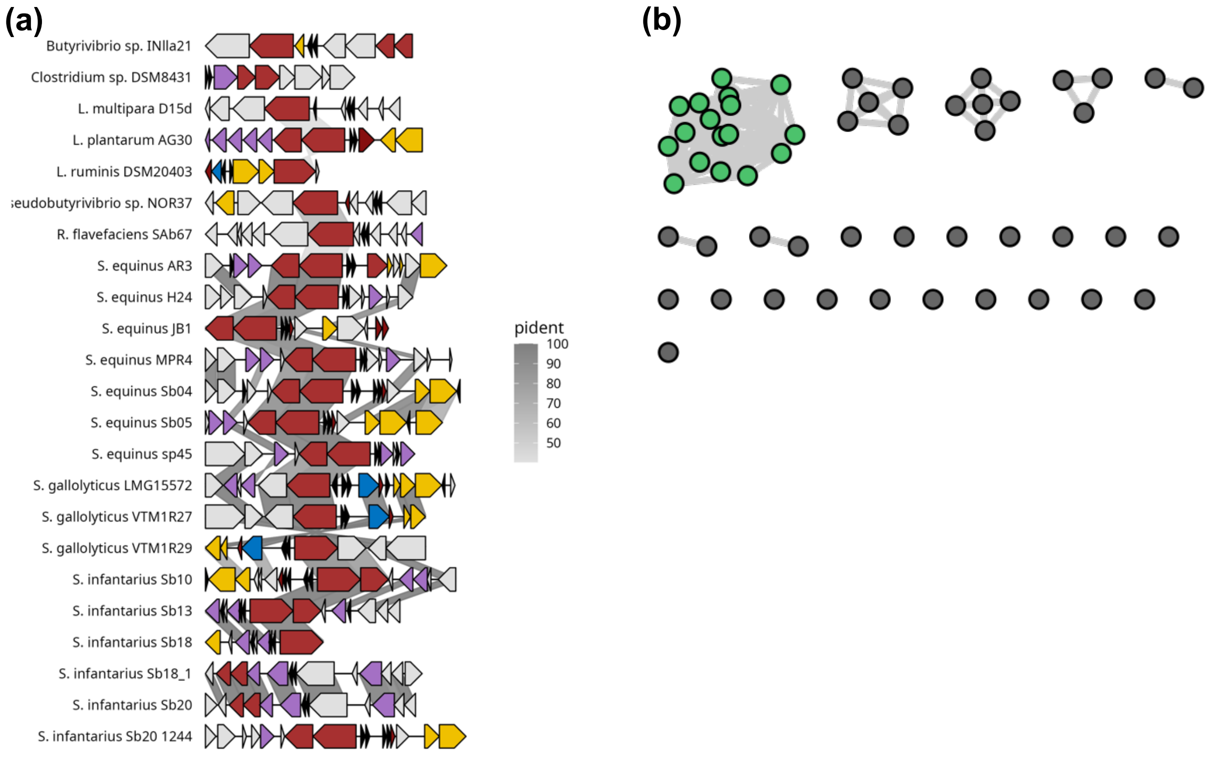

Figure S4. Class IIb bacteriocins (a) Operons encoding class IIb two-component unmodified bacteriocins in the Hungate1000 culture collection. **(b)** Sequence similarity network of core peptides predicted showing high degrees of variation between core peptides. The green cluster represents peptides dominantly in Streptococcus sp. and are clustered due to conserved leader sequences between alpha and beta peptides.

In silico identification of bacteriocin gene clusters in the Hungate1000 ruminant prokaryote culture collection.

Additional biosynthetic gene clusters and associated core peptide alignments are provided in below.

Ranthipeptides

The ranthipeptides (radical non-α-carbon thioether peptides) are a recently reported class of RiPP characterised by the presence of a Cys-rich precursor peptide. They are locally encoded with a radical S-adenosylmethionine (rSAM)-dependent enzyme (PF04055). 217 putative ranthipeptides were identified within the Hungate1000. Core peptides with a six-cysteine peptide SCIFF and a putative transport mechanism within the BGC were considered ranthipeptides, providing these were on the same strand as the Radical_SAM. SCIFFs were found ubiquitously among *Clostridia* within the Hungate and are similar to freyrasin (45).

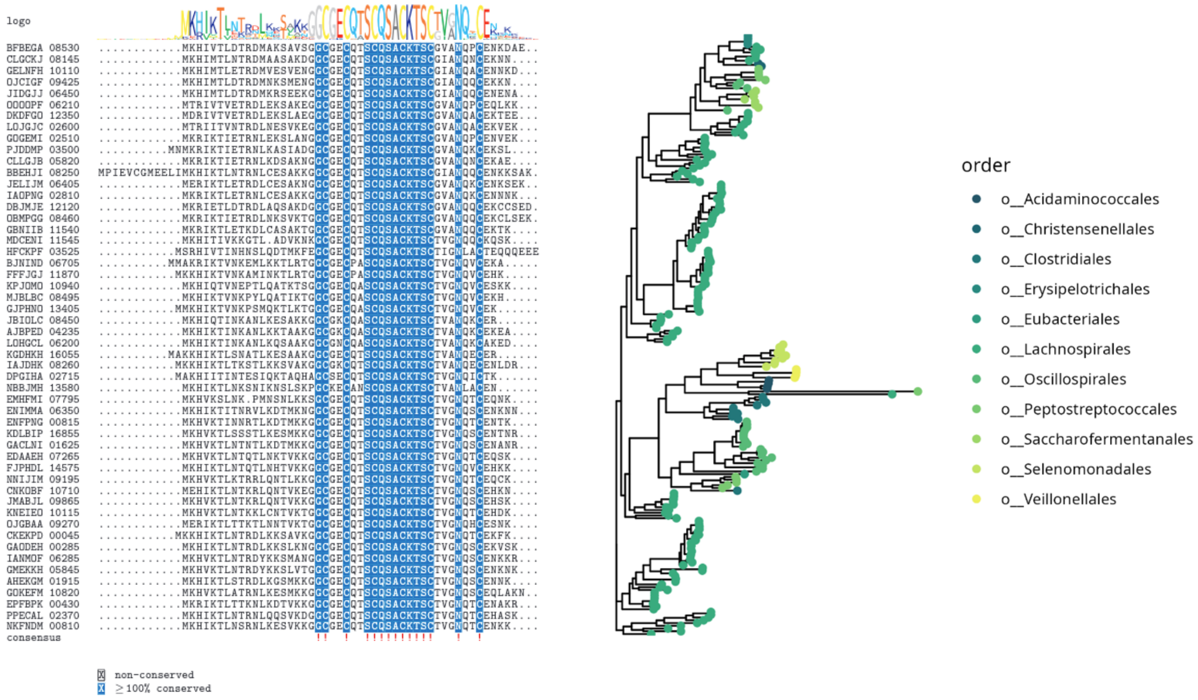

Ranthipeptide core peptides (a) Alignment of a reduced set of ranthipeptide core peptides**.** Core peptides with six-cysteine residues were clustered using cd-hit at a 90% threshold and highlighted are residues that are 100% conserved. **(b)** An unrooted phylogenetic tree of all ranthipeptide core peptides with 100 bootstrap replicates.

Thiopeptide

Thiopeptides are a group of highly modified, sulphur-rich, potent, peptide antibiotics often with activity against clinically relevant pathogens including Methicillin-resistant *Staphylococcus aureus* and *Clostridium difficile*. The core peptides, often part of a large operon (up to 32kb), contain their own characteristic modified structure, rich in cysteine, threonine and serine residues which are capable sites for dehydration modification. A repertoire of modification proteins is responsible for dehydration, dehydrogenation, ring formation and deamination of these structures, and they often contain homology to modification proteins in other classes of BGCs, notably the LazF

gene is composed of the N-terminal region of a lantibiotic dehydratase domain in the production of lactazole RiPPs, one of the shortest operons for thiopeptide synthesis (77). Thiopeptides exert their antimicrobial effect through inhibition of protein synthesis or RNA polymerase, with the former being the more frequently distributed mechanism of action. Thiostrepton is a 26-atom macrocyclic peptide that binds to the 23S rRNA and L11 protein within the 50S subunit of the ribosome of susceptible bacteria and has been used in the veterinarian setting to treat mastitis. Recently the thiopeptide field has expanded upon known gene clusters thanks to *in silico* mining, the majority of which 95.1% are among Actinobacteria or Bacilli classes (78). Thiopeptides have multiple ecological roles within the microbiome including interference competition, stimulation of biofilm formation and cell morphological development (46). Ten putative thiopeptide BGCs were predicted within the Hungate1000 and their predicted core peptides are in Table S4.

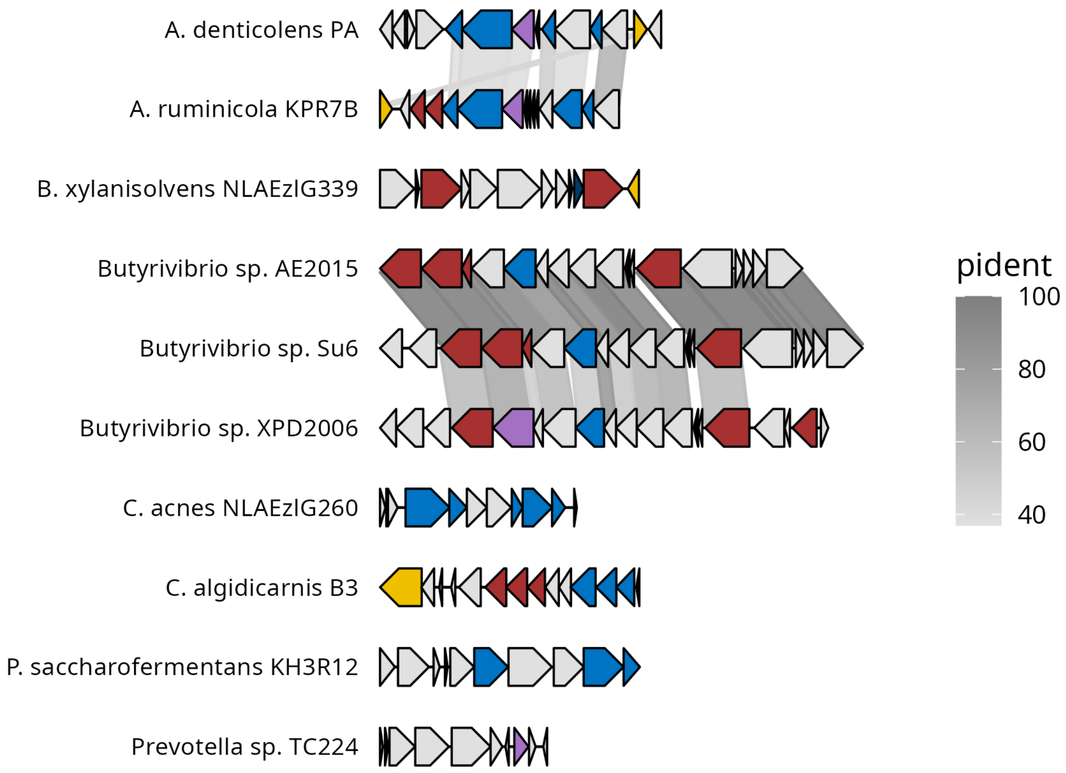

Operons encoding thiopeptide BGCs in the Hungate1000.

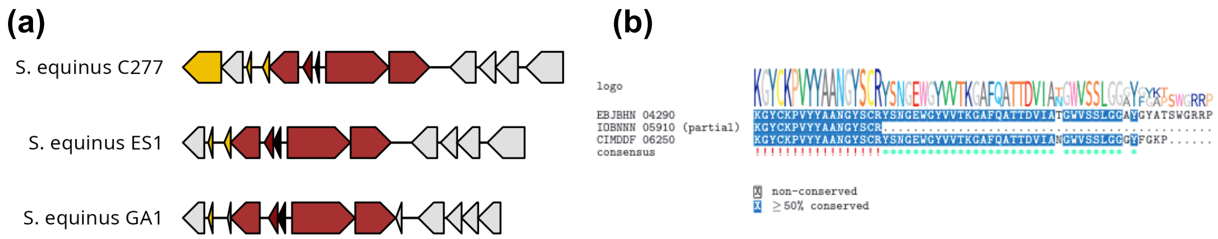

Angicin-like BGCs (a) Angicin BGCs present in *S. equinus* strains from the Hungate1000. **(b)** Angicin-like core peptides. *S equinus* C277 encodes a partial angicin-like core peptide.

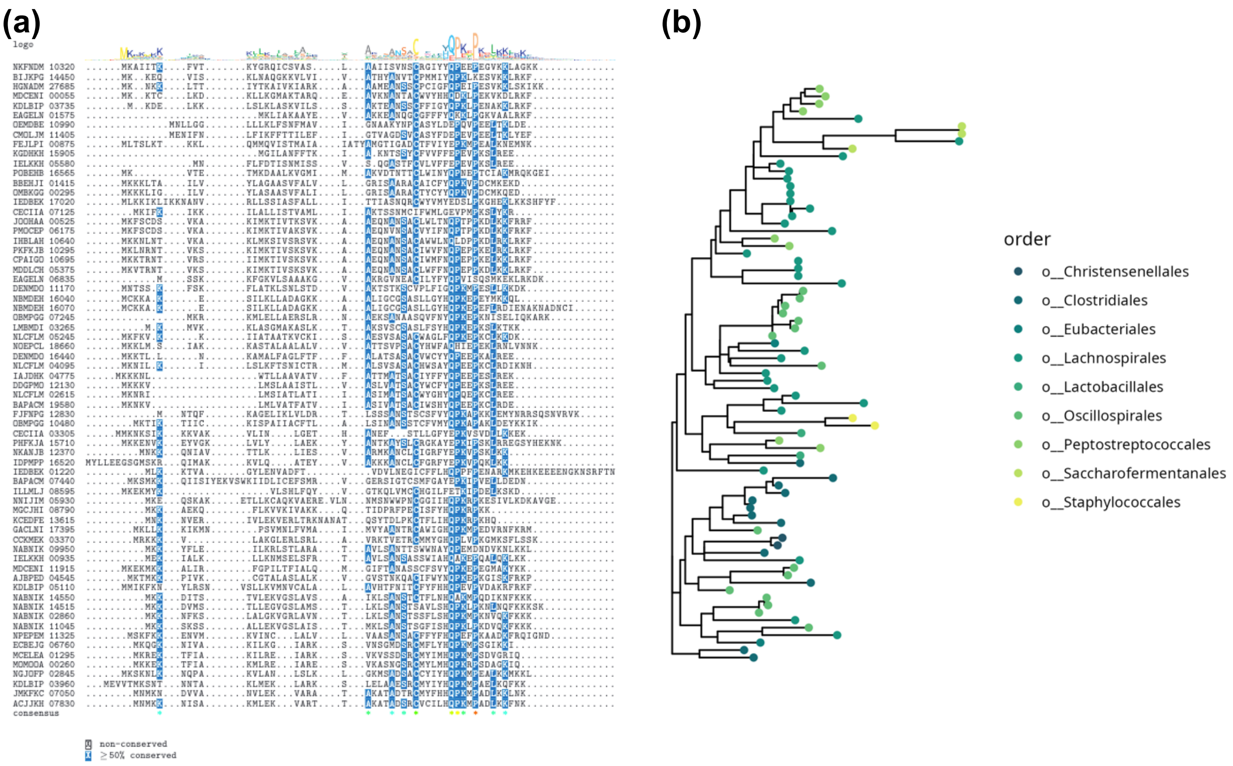

Cyclic lactone auto inducer peptides (a) Sequence alignment of cyclic-lactone-autoinducing peptides from the Hungate1000. Of note is a highly conserved “PK” residues that are also conserved in Auto_Inducing_Peptide_IV from *Staphylococcus epidermidis* (WP_001094303.1). **(b)** Proteins matching the domain TIGR04223 from antiSMASH were used to construct the tree. These proteins resemble AgrD, the 46 amino acid peptide with cross-reactivity among Staphylococcus spp. (79).
