# Supplementary material for: The Hungate1000 prokaryotic culture collection encodes a wide variety of bacteriocins
Source: mSystems. 2026 May 27;11(6):e00195-26. doi: 10.1128/msystems.00195-26 (PMC13289728; doi:10.1128/msystems.00195-26)
Supplement: Table S6 — HTML-formatted version .tsv table on Zenodo. [file msystems.00195-26-s0008.html]

GMSC Samples Metadata - Interactive Table 


# GMSC Samples Long Metadata (Trial)

Interactive table with search and sorting - All 40,789 rows

Loading and decompressing data...

10 per page
25 per page
50 per page
100 per page

No results found
